# Literature Review Comparing Laparoscopic and Percutaneous Endoscopic Gastrostomies in a Pediatric Population

**DOI:** 10.1155/2010/507616

**Published:** 2010-03-10

**Authors:** Madelen Lantz, Helena Hultin Larsson, Einar Arnbjörnsson

**Affiliations:** Department of Pediatric Surgery, Lund University Hospital, Sweden and Lund University Medical Center Skane (UMCS), 221 85 Lund, Sweden

## Abstract

*Objective*. This study compares laparoscopic and percutaneous endoscopic gastrostomy (PEG) in a paediatric population to test the hypothesis that there is a difference in the frequency of serious gastrointestinal complications between the two methods. *Methods*. All reports published between 1995 and 2009 on laparoscopic gastrostomy and PEG in children was included. Prospective and retrospective trials, comparing the two methods or dealing with one of them only were included. Endpoints were accidentally performed gastrointestinal fistula causing an emergency re-operation. The frequency of inadvertent gastroenteric fistulas using the two different techniques was calculated. *Results*. 822 publications were found when using the search terms: gastrostomy, gastrointestinal complications, and all child: 0–18 years. From these, 54 studies were extracted for this investigation. These studies reported a total of 4331 children undergoing gastrostomy operation, 1027 by using the laparoscopic technique and 3304 using the PEG technique. The number of serious gastrointestinal fistulas to colon or small bowel was 0% and .27%, respectively, *P* < .05. *Conclusions*. The results suggest that by performing laparoscopic gastrostomy in children it is possible to avoid the serious intestinal fistula complications caused by a blind puncture through the abdominal cavity when performing the PEG.

## 1. Introduction

The open surgical placement of a gastrostomy tube in children may cause significant complications, such as wound infection, leakage, and excessive granulation tissue. The use of a percutaneous gastrostomy tube (PEG) obviates the need for a laparotomy [1, 2]. The technique is associated with the same complications, along with the risk of inadvertent bowel injury due to a blind puncture through the abdominal cavity [3–5]. To avoid the complications associated with the PEG technique, laparoscopic-assisted gastrostomy has been used at our centre since 1994, avoiding the risk of bowel injuries [6–12].

 This study aims to use a literature review to compare the frequency of serious gastrointestinal complications after laparoscopic versus percutaneous endoscopic gastrostomy (PEG) in a pediatric population. The specific question that our study intends to answer is: Does the laparoscopic procedure significantly reduce the rate of gastrointestinal complications of fistula that necessitated operation compared with PEG? We are not aware that any literature review of gastrostomy complications in a pediatric population has been performed previously to answer that question.

## 2. Method

A systematic review of articles published in the English and Swedish languages between 1995 through November 2009 was performed by searching the following electronic databases: Pub Med, Web of Science and Cochrane Library. Randomized controlled trials, case series reports, clinical trials, practice guidelines, meta-analysis, reviews and editorials were searched for. 

The detailed search strategy included the terms:

- gastrostomy, percutaneous endoscopic gastrostomy, laparoscopic gastrostomy, video-assisted gastrostomy,
- gastrointestinal complications, gastrocolic fistula, gastroenteric fistula,
- children,
- ages: all child: 0–18 years.

Data for the following outcomes were extracted and included in the literature review of the study.

- Reports on children undergoing the PEG or the VAG method for a gastrostomy.
- Studies where the gastrostomy is performed as the sole operative intervention.
- Studies where peri- and postoperative complications are reported.
- Reports on gastrointestinal fistula caused by the operative intervention.
- Gastrointestinal fistula requiring reoperation of the patient.
- Surgery-related mortality and morbidity including: intra-abdominal bleeding, bowel injury, wound infection causing sepsis, and intra-abdominal infection.

We excluded

- surgery-related morbidity noted after discharge from the hospital correlated to the fact that the child had been operated on and correlated to the child's general condition, including prolonged hospital stay and readmission reviewed by a doctor;
- complications and morbidity correlated to the fact that the child had got a foreign body through their abdominal wall into the stomach, not depending on the type of gastric tube or the method used for the operation, including pain, nausea, vomiting, granuloma, leakage, infection demanding the use of antibiotics.

### 2.1. Statistical Method

For statistical calculation the *F* test was used. A *P*-value <.05 was considered significant. All statistical computations were made using SPSS version 15 (SPSS, Inc., Chicago, IL, USA).

## 3. Results

Meta-analysis, randomized trials, or comparative studies were not found. 822 case series reports publications on gastrostomy in children were identified; 54 filled the criteria of the study and were included. The results are summarized in Table 1 disclosing that unintentional damage to the colon or small bowel is reported after 1.27% of the PEG operations only, and this is caused by the blind puncture through the abdominal cavity. The difference is significant: *P* < .05.

## 4. Discussion

This study has investigated the possible benefits of VAG over PEG in a pediatric population, where reduced postoperative complications are particularly desirable. The results of this literature review of 54 studies, retrospective and prospective, suggest that postoperative gastrointestinal complications are statistically significantly reduced or nonexistent in children undergoing VAG compared with PEG. This is in accordance with the previous studies reporting a 2%-3% frequency of gastrointestinal fistulas when using the PEG method in children [2, 13]. The results suggest that gastrointestinal postoperative complications are more likely to be fewer with the use of VAG than with PEG. We have no reason to believe that the frequency of a postoperative wound infections or postoperative intestinal obstruction due to adhesions differs between these two groups.

Reviewing the literature we found a total of 4331 subjects, of which 3304 (76%) underwent PEG and 1027 (24%) underwent VAG. A sample group this size would otherwise be impossible to accumulate in a reasonable length of time in a randomized control trial. These findings should, however, be treated with caution because of the lack of randomized comparative trials of PEG versus VAG in the pediatric population available for use in the meta-analysis. 

A literature review can be used to evaluate the literature in both qualitative and quantitative ways by comparing and integrating the results of different studies, accounting for variations in characteristics that can influence the overall estimate of outcomes of interest. It is important, however, to address the limitations of the literature review, which were as follows:

- First: different studies may have had slightly different defining criteria for the outcome measures we were interested in. This would not apply to the complications in need of operative interventions which were relatively homogeneously reported throughout the studies. In this paper, every attempt is made to select outcome measures that are as absolute as possible, such as the gastrointestinal complications registered here, in order to reduce heterogeneity.
- Second: neither the allocation of treatment, that is, type of operation, nor the assessment of outcome was blinded. This paper is based on clinical reports from centers using only one of the methods studied. A meta-analysis of randomized controlled trials would have been preferred for answering the question posed here, but these are still missing.
- Third: it is important to bear in mind nonpublication bias, particularly in a review based on published studies. The studied material is, moreover, biased by the fact that some of the authors do not report their results. Thus we have probably missed a considerable number of operated on children as well as several complications that have not been reported.
- Fourth: there was variation in inclusion criteria, study type, type of randomization, treatment protocols, and outcome assessment between studies.
- Finally: although the studies included in this paper reported results on a paediatric population, there were patient groups who represented a population that was in the wide paediatric age range from newborns to 18 years old.

Apart from these limitations, we think that an important link has been identified between VAG and a reduction in gastrointestinal complications compared with PEG in a pediatric population. In addition to helping to answer the question of whether VAG reduces gastrointestinal complications compared with the PEG procedure, this study raises several important issues regarding the factors that need to be taken into account when comparing the two surgical techniques. This could become evident in a sensitivity analysis, which may show the level of heterogeneity to be the lowest with studies that are of randomized design, followed by prospective studies. To further reduce heterogeneity, studies must be of an adequate size, and older publications may increase heterogeneity because surgical technique, degree of proficiency, and types of instrumentation can all change significantly over a period of 15 years. 

It is important to note that there are more factors that should be matched when comparing PEG and VAG groups. We did not register concomitant diseases, whether the children are operated on at a pediatric surgical centre or in a general surgical unit, with or without university affiliation. Some of the major advantages of VAG are the siting of the gastrostomy to allow for later fundoplication, which is necessary in up to 20% of patients, without takedown of the previously placed gastrostomy. Furthermore, primary placement of a balloon button, instead of the hard PEG disc, is preferred since the removal of the PEG disc usually requires an anesthetic in small children whereas changing a balloon button is simple and can be done in an office setting without analgesics or sedation. Another advantage is that one does not have to bring more equipment into the room for the PEG gastrostomy after laparoscopic fundoplication.

 Although this study adds weight to the argument that video-assisted or laparoscopic gastrostomy results in fewer preoperative gastrointestinal complications when compared with the percutaneous endoscopic gastrostomy, it is important to appreciate that it does not attempt to evaluate the different minimally invasive surgical techniques. The analysis further highlights the need for high-quality randomized trials, comparing PEG to VAG in pediatric patient groups matched not only for age and sex, but also for weight and concomitant diseases. Finally, results such as those produced by our study may lead to more accurate informed parental consent when explaining the risks of PEG versus VAG in children.

This paper presents a subject that is very relevant to pediatric surgeons because it addresses one of the most commonly done procedures in daily practice. However, this is a raw data analysis of a very heterogeneous group of papers with a very uneven numbers of patients in each group. The analyzed papers were not stratified as to their purpose, as to who did the procedures (surgeons, gastroenterologists, radiologists, others) and their level of experience, what special anatomical problems the different patients had (previous operations, scoliosis, or very young age), for indications for the gastrostomy as well as other factors. Radiologically placed gastrostomies are included although technically these are not PEGs. 

 It is quite logical that, during a laparoscopic procedure, where the surgeon is looking at the stomach, injury to the bowel is unlikely. Not included in the discussion are the complications associated with the laparoscopic interventions, as well as their cost. In future work emphasis should be on the problems encountered with each one of the techniques and how to best avoid them.

## Figures and Tables

**Table 1:** A summary of the reports found in the literature describing complications after minimally invasive gastrostomy in children using two different techniques, Percutaneous Endoscopic Gastrostomy (PEG) or Video-Assisted Gastrostomy (VAG) or laparoscopic gastrostomy.

	Percutaneous Endoscopic Gastrostomy PEG	Video-Assisted Gastrostomy VAG	Statistics*
Number of children reported, *n* = 4331	3304	1027	
Number of gastrointestinal complications	42(1, 27%)	0	*P* < .05*
Number of publications, four publications reported both PEG and VAG. Total: 54 publications	31	19	

*Statistical method: *F* test.
